# Glioma stem cells and neural stem cells respond differently to BMP4 signaling

**DOI:** 10.1186/s13619-022-00136-5

**Published:** 2022-11-01

**Authors:** Xin-Xin Han, Chunhui Cai, Li-Ming Yu, Min Wang, Wenhan Yang, Dai-Yu Hu, Jie Ren, Lu-Ying Zhu, Jia-Jia Deng, Qing-Qing Chen, Hua He, Zhengliang Gao

**Affiliations:** 1 Shanghai Key Laboratory of Craniomaxillofacial Development and Diseases, Shanghai Stomatological Hospital & School of Stomatology, Fudan University, Shanghai, 200001 China; 2 Yangzhi Rehabilitation Hospital (Shanghai Sunshine Rehabilitation Center), Tongji University School of Medicine, Shanghai, 200072 China; 3 Institute of Geriatrics (Shanghai University), Affiliated Nantong Hospital of Shanghai University (The Sixth People’s Hospital of Nantong), School of Medicine, Shanghai University, Nantong, China; 4 School of Medicine, Jiaxing University, Jiaxing, China; 5 Department of Neurosurgery, Changzheng Hospital, Second Military Medical University, Shanghai, China; 6 Department of Neurosurgery, Third Affiliated Hospital of Second Military Medical University, Shanghai, China

**Keywords:** Glioma stem cells, BMP4, Neural stem cells, Cell differentiation, Cell proliferation

## Abstract

Malignant glioma is a highly heterogeneous and invasive primary brain tumor characterized by high recurrence rates, resistance to combined therapy, and dismal prognosis. Glioma stem cells (GSCs) are likely responsible for tumor progression, resistance to therapy, recurrence, and poor prognosis owing to their high self-renewal and tumorigenic potential. As a family member of BMP signaling, bone morphogenetic protein4 (BMP4) has been reported to induce the differentiation of GSCs and neural stem cells (NSCs). However, the molecular mechanisms underlying the BMP4-mediated effects in these two cell types are unclear. In this study, we treated hGSCs and hNSCs with BMP4 and compared the phenotypic and transcriptional changes between these two cell types. Phenotypically, we found that the growth of hGSCs was greatly inhibited by BMP4, but the same treatment only increased the cell size of hNSCs. While the RNA sequencing results showed that BMP4 treatment evoked significantly transcriptional changes in both hGSCs and hNSCs, the profiles of differentially expressed genes were distinct between the two groups. A gene set that specifically targeted the proliferation and differentiation of hGSCs but not hNSCs was enriched and then validated in hGSC culture. Our results suggested that hGSCs and hNSCs responded differently to BMP4 stimulation. Understanding and investigating different responses between hGSCs and hNSCs will benefit finding partner factors working together with BMP4 to further suppress GSCs proliferation and stemness without disturbing NSCs.

## Background

Malignant glioma is a brain cancer with high lethality and one of the most extensively genetically characterized cancers (Wu and Mischel 2020). The median overall survival is only approximately 14 months with radiotherapy plus temozolomide treatment, despite a large amount of genomic, methylation, and therapy data available from hundreds of clinical samples (De Boeck et al. 2020; Perry et al. 2017). Surgery, radiation therapy, chemotherapy, and targeted agents have all been employed to treat glioma; however, these traditional strategies showed poor improvement in survival and long-term toxic effects on the central nervous system (CNS) and bone marrow (De Boeck et al. 2020; Desjardins et al. 2018). At the same time, malignant glioma has a high recurrence rate, leading to neurocognitive function loss and a decrease in health-related quality of life (Chinot et al. 2014). The median recurrence period is approximately 7 months, and death normally occurs 7 months later (Couturier et al. 2020). The high recurrence rate of malignant glioma was due to the existence of glioma stem cells (GSCs) from the tumor environment, which is known to be responsible for their high resistance to radiotherapy and chemotherapy (Couturier et al. 2020; Neftel et al. 2019; Zhu et al. 2020). To improve the treatment of malignant glioma, identifying prognostic biomarkers from whole genomic analysis or novel agents specifically targeting GSCs has become popular recently (Cho et al. 2013; Tang et al. 2020). The key to GSCs therapy is to inhibit the resistance ability of GSCs during chemotherapy and radiotherapy but keep the safety of normal NSCs. Thus, a deep understanding of the differences between hGSCs and hNSCs can provide more evidence for selecting valid therapeutic targets.

Bone morphogenetic protein4 (BMP4) belongs to BMPs signaling. Besides playing as an osteoinductive factor in bone development, BMP4 is also well studied as a key player regulating neuronal development in the embryonic, postnatal, and injured central nervous system (Cole et al. 2016). In neural stem cells, BMP4 was reported to induce stem cell quiescence both in vivo and in vitro through the Wnt signaling pathway (Marques-Torrejon et al. 2021; Sun et al. 2011). For NSCs differentiation, BMP4 showed positive roles in promoting astrocyte differentiation but abrogated oligodendrogliogenesis (Cole et al. 2016).

Similar in hGSCs, BMP4 also shown ability to drive astroglia differentiation to inhibit tumor cell proliferation (Liu et al. 2010; Piccirillo et al. 2006; Piccirillo and Vescovi 2006). During the last decade, BMP4 has been involved in several clinical treatments to improve the survival of GBM patients. The expression of BMP4 is higher in low-grade gliomas, with lower mortality rates as compared to high-grade gliomas (Nayak et al. 2020). It has been considered a prognostic marker for adult gliomas (Bao et al. 2013; Wu and Yao 2013). However, the functional role of the BMP signaling in GSCs’ resistance to chemotherapy or radiotherapy remains controversial. Some groups reported that the inhibition of BMPs only decreases cell growth but cannot affect the stemness of GSCs (Sachdeva et al. 2019) while others indicated that BMP4 could reduce glioma stemness by inducing their differentiation (Nayak et al. 2020). Expect the study of BMP4 individual function in GSCs, the study of BMPs and other pathways crosstalk shown improvement in chemotherapy resistance. For example, BMP4-induced differentiation increases the temozolomide sensitivity of GSCs expressing high levels of epidermal growth factor receptor (EGFR) (Ciechomska et al. 2020). Thus, transcriptomics study after BMP4 treatment in GSCs may help shed light on finding efficient partner factors working together with BMP4 in further clinical application. Multiple BMP4 delivery strategies have been recently designed to overcome its short half-life time in vivo (Calpe et al. 2016; Olmsted et al. 2003). Among them, hNSCs-based delivery can transfer the therapeutic agent to malignant growth sites with their great migratory capacity (Kendall et al. 2008; Schmidt et al. 2005). The homing ability of hNSCs, together with BMP4 expression, can decrease GSCs’ growth ability both in vitro and in vivo (Liu et al. 2016). In this way, it is attractive to study and analyze the different gene changes after BMP4 stimulation in GSCs and NSCs. The genes stable expressed in hNSCs but significantly upregulated or downregulated in hGSCs could be considered as markers to predict the outcome of hNSCs-based delivery BMP4 treatment.

In this study, we used isolated hGSCs from surgical samples of glioma patients and the established hNSC lines to study their responses to BMP4 treatment (Han et al. 2017; Han et al. 2021). First, we observed the morphological changes in cells after BMP4 stimulation. BMP4 significantly inhibited the proliferation rate in hGSCs but did not affect cell growth in hNSCs. At the same time, we collected RNA samples for transcriptional analysis to further study the underneath signaling responses in these two types of cells. The Differential Expression Analysis (DEG) identified four groups of upregulation and downregulation genes both in hGSCs and hNSCs. We compared these gene lists, and selected genes only changed in hGSCs. The KEGG analysis showed that cell proliferation and cell differentiation signaling pathways were enriched. Based on the guidance of detailed gene lists, we performed immunostaining assays to validate the expression of certain protein makers in hGSCs, such as S100-beta, SOX2, and Ki67. Our results offered detailed information on the BMP4-mediated inhibitory effects on hGSCs. This study will aid in the understanding of the differential responses of hGSCs and hNSCs to BMP4. The increased understanding may benefit drug screening and therapy for glioma treatment.

## Results

### BMP4 suppressed the growth of hGSCs and increased the body size of hNSCs but not hGSCs

**Fig. 1:**
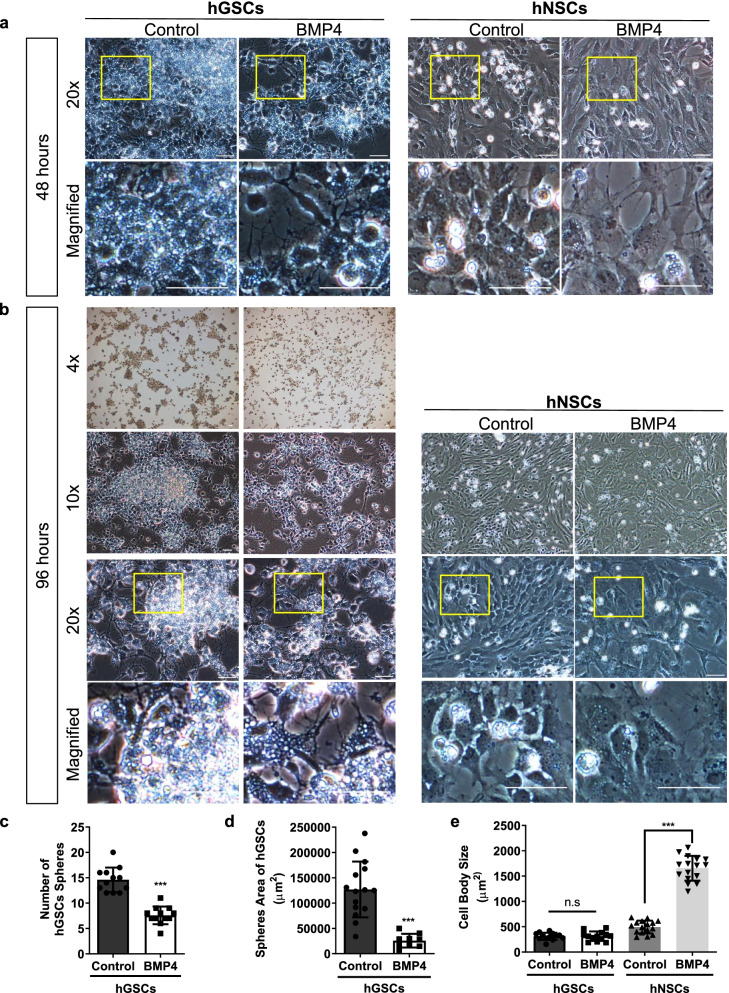
Bone morphogenic protein 4 (BMP4) inhibits the growth of human glioma stem cells (hGSCs) and changes the body size of human neural stem cells (hNSCs). **a** Schematic representation showing hGSCs treated with 0 ng/mL (Control) or 20 ng/mL BMP4 for 48 h. Left: hGSCs; right: hNSCs. The yellow box represents the source area of the magnified image. **b** Schematic representation showing hGSCs treated with 0 ng/mL (Control) or 20 ng/mL BMP4 for 96 h. Left: hGSCs; right: hNSCs. The yellow box represents the source area of the magnified image. **c** Quantitative analysis of hGSC sphere numbers measured using ImageJ. **d** Coverage areas of single hGSC spheres were calculated. **e** The body size of both hGSCs and hNSCs was observed and measured using ImageJ (× 4, × 10, and × 20 magnification). Scale bar, 50 μm. Data are presented as means ± SD. Student’s t-test. n.s., not significant; ∗∗∗*p* < 0.001

To compare the differences between hGSCs and hNSCs after BMP4 stimulation, we first added the BMP4 factor into the basic medium (DMEM/F12 supplemented with N-2, B-27, GlutaMAX, penicillin, and streptomycin) containing 10 ng/mL FGF. Two groups were designed as control without BMP4 treatment and BMP4 group with BMP4 treatment both in hGSCs and hNSCs. Each group of cells was seeded at the same density at the beginning for 16 hours, then BMP4 (50 ng/ml) was added to the BMP4 treatment group. Photos were taken every 24 hours to observe the cell growth. After BMP4 stimulation for 48 hours, hGSCs showed decreased cell density and unchanged body size, whereas the body size of hNSCs increased (Fig. 1a). After 96 h, the above phenotypes were more significant. The number of cells in the BMP4 treatment group was less than the control group in hGSCs, while the cell size increased in hNSCs (Fig. 1b).

We used Image J to analyze the cell culture results quantitatively. For hGSCs, there are two morphologies of cells in sphere state and non-sphere state. We can observe both sphere area and non-sphere area at the same plate. The cell body size was measured with single cells having clear edges. The number of hGSCs spheres was counted with low-power field (4x) pictures, and the size of hGSCs spheres was measured with Image J. Statistical. The analyses showed that the number of hGSCs spheres (Fig. 1c) and the area of cell spheres (Fig. 1d) were significantly reduced after BMP4 treatment. In contrast to that observed with hNSCs, BMP4 treatment did not change the body size of hGSCs (Fig. 1e).

### Transcriptional changes in hGSCs and hNSCs after BMP4 stimulation

**Fig. 2:**
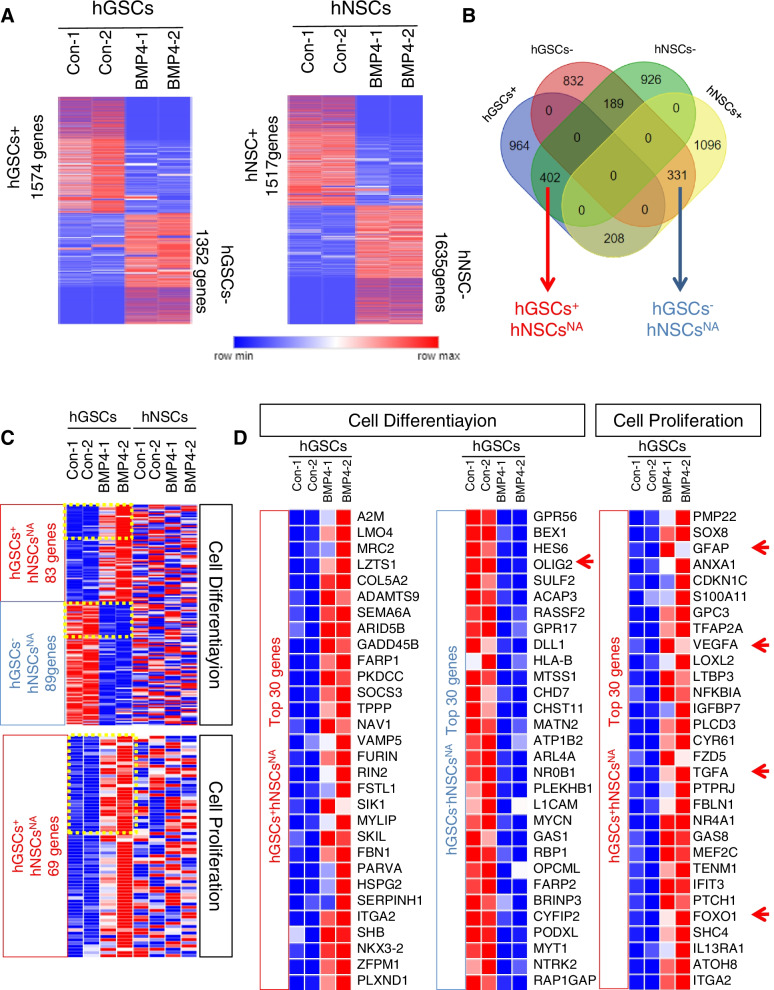
Transcriptional changes in human glioma stem cells (hGSCs) and human neural stem cells (hNSCs) after BMP treatment. **a** Gene clustering from control (Con-1 and Con-2) and BMP treatment (BMP-1 and BMP-2) in hGSCs/hNSCs RNA sequencing results show significantly upregulated and downregulated genes as hGSCs^+^ (1574 genes), hGSCs^−^ (1352 genes), hNSCs^+^ (1517 genes) and hNSCs^−^ (1635 genes). **b** The Venn diagram shows the overlap of 4 gene lists in (**a**) to identify hGSCs-hNSCsNA (331 genes) and hGSCs+hNSCsNA (402 genes) lists. **c** Cell differentiation and cell proliferation pathways were identified in Gene Ontology analysis of hGSCs and hNSCs gene list. Expression patterns of hGSCs^+^hNSCs^NA^ (83 genes)/hGSCs^−^hNSCs^NA^ (89 genes) in cell differentiation and hGSCs^+^hNSCs^NA^ (69 genes) in cell proliferation were shown as heatmap. **d** Detailed expression patterns of top 30 genes in hGSCs^+^hNSCs^NA^ and hGSCs^−^hNSCs^NA^ were shown as heatmap

BMP4 has been reported to play an important role both in the quiescence and the differentiation of hGSCs (Ciechomska et al. 2020; Sachdeva et al. 2019; Videla Richardson et al. 2016). BMP4 may also promote the differentiation and increase the invasiveness of human neural progenitor cells (Duval et al. 2019; LaVaute et al. 2009; Sailer et al. 2013; Weible and Chan-Ling 2007). However, how BMP4 affects hGSCs and hNSCs has not been systematically analyzed. Elucidating the similarities and differences is critical for finding drugs or therapies that can inhibit hGSCs but not normal nerve tissue cells. Consequently, we sought to analyze the differential responses between the two cell types after BMP4 treatment. We performed RNA sequencing in hGSCs and hNSCs with or without BMP4 treatment. In order to collect curate genetic information, we collected RNA samples from Passage 10 hGSCs and Passage4 hNSCs. Two biological replicates were provided for each group. Using different expression gene analysis with BMP4 stimulation in hGSCs, we identified 1574 genes as upregulated genes and 1352 genes as downregulated genes. In hNSCs, 1517 genes were upregulated and 1635 downregulated following exposure to BMP4 (Fig. 2a). Overlapping analysis of these four groups of genes was performed to select genes only changed in hGSCs. Three hundred thirty-one genes were named as hGSCs^−^hNSCs^NA^ while 402 genes were named as hGSCs^+^hNSCs^NA^ (Fig. 2b). Then, we performed KEGG and Gene ontology analysis within these genes. Cell differentiation and cell proliferation pathways related genes were enriched (Fig. 2c). Interestingly, the genes, which identified as being involved in cell differentiation, showed both upregulation (83 genes) and downregulation (89 genes) following BMP4 stimulation (Fig. 2c). The cell proliferation pathway corresponding genes (69 genes) only increased in hGSCs but disordered in hNSCs. Detailed expression patterns of the top 30 genes in hGSCs+hNSCsNA and hGSCs-hNSCsNA are shown as a heatmap (Fig. 2d).

**Fig. 3:**
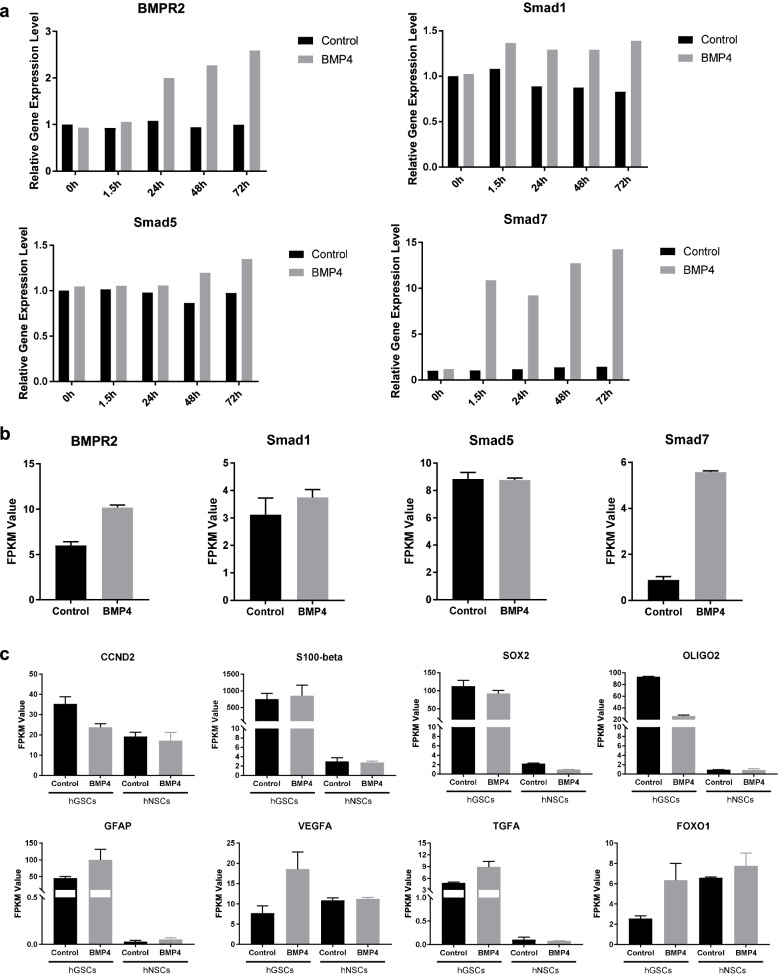
Q-PCR results and FPKM values of candidate genes. **a** Q-PCR results of BMP4 signaling downstream response genes. **b** FPKM value of BMP4 signaling downstream response genes. **c** FPKM value of other candidate genes identified from hGSCs^+^hNSCs^NA^ and hGSCs^−^hNSCs^NA^ KEGG analysis

We selected several candidate genes and highlighted them with red arrows shown in Fig. 2d. To validate whether FPKM value stands for gene expression level, we performed a Q-PCR experiment and compared trends of the gene with FPKM value. BMP4 signaling downstream response genes BMPR2, Smad1, Smad5, and Smad7, showed similar trends after BMP4 stimulation in hGSCs (Fig. 3a-b). The expression level of CCND2, S100-beta, Sox2, OLIG2, GFAP, VEGFA, TGFA, and FOXO1 in hNSCs and hGSCs are presented in the bar chart (Fig. 3c). These genes only changed in hGSCs could be considered as candidate genes for BMP4 combination partner factor in future to improve the inhibition of tumorigenesis.

### BMP4 treatment reduced the levels of S100-beta and Sox2 in hGSCs

**Fig. 4:**
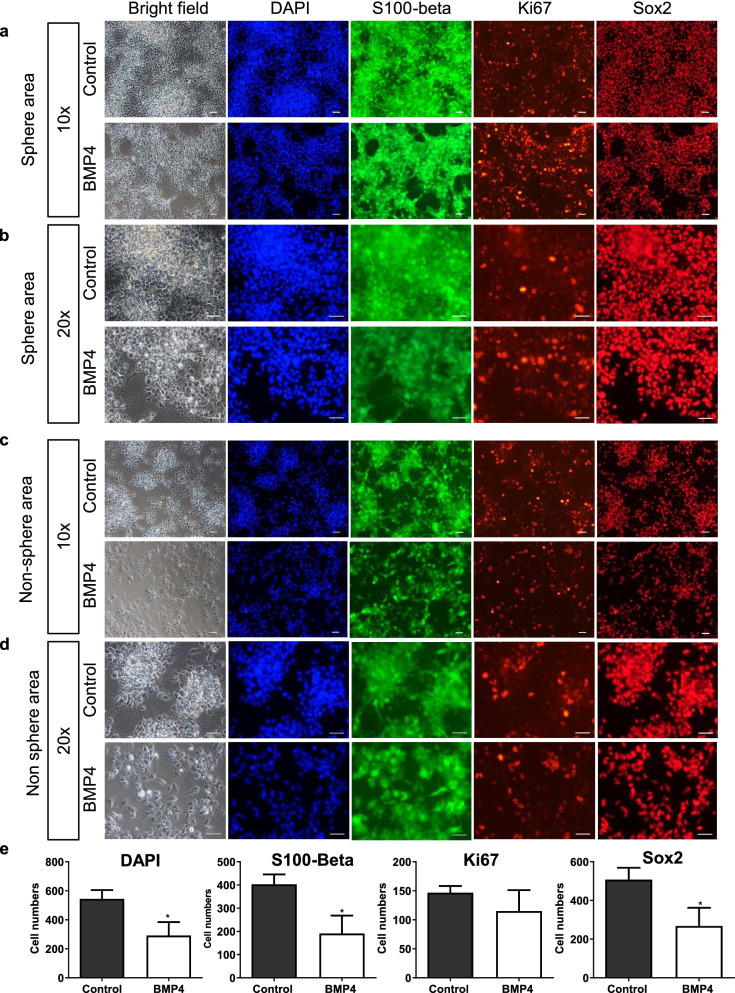
BMP4 reduced S100-beta positive, Sox2 positive and DAPI staining in hGSCs, but no significant decrease in Ki67 positive cells. **a** Representative image of the sphere area of hGSCs immunostained for S100-beta, Ki67, Sox2, and DAPI (nuclei). Control (untreated) and BMP4-treated (20 ng/mL) hGSCs were fixed and stained after 6 days of treatment. **b** Representative images of (a) at higher magnification (control and BMP-treated [20 ng/mL]). **c** Representative images of nonsphere area hGSCs immunostained for S100-beta, Ki67, Sox2, and DAPI (nuclei). **d** Representative images of (c) at higher magnification. **e** Quantitative analysis of DAPI, Ki67, S100-beta, and Sox2 expression in BMP4-stimulated hGSCs measured using ImageJ. Data are presented as means ± SD. Student’s t-test. ∗*p* < 0.05

The results of KEGG analysis indicated that genes involved in cell differentiation and proliferation were differently expressed between hGSCs and hNSCs. We then used immunofluorescence staining to further examine the effects of BMP4 treatment on cell numbers and the expression of S100-beta, Ki67, and Sox2 on day 6 after BMP4 stimulation. Depending on the cell morphology after BMP4 stimulation in hGSCs, we classified the cell pictures into sphere areas and non-sphere areas. The expression of S100-beta stands for the differentiation ability of cells, while Ki67 stands for the proliferation rate of cells. The low-power field (Fig. 4a) and high-power field (Fig. 4b) of the area where the cell spheres with relatively concentrated were detected. The low-power field (Fig. 4c) and high-power field (Fig. 4d) of the area with relatively few cell spheres were also detected. We found that BMP4 treatment reduced the total number of cells by counting the number of DAPI staining. However, Ki67 expression did not decrease, and S100-beta/Sox2 expression showed only a slight decrease (Fig. 4e).

### Changes in the ratios of marker genes after BMP4 treatment

**Fig. 5:**
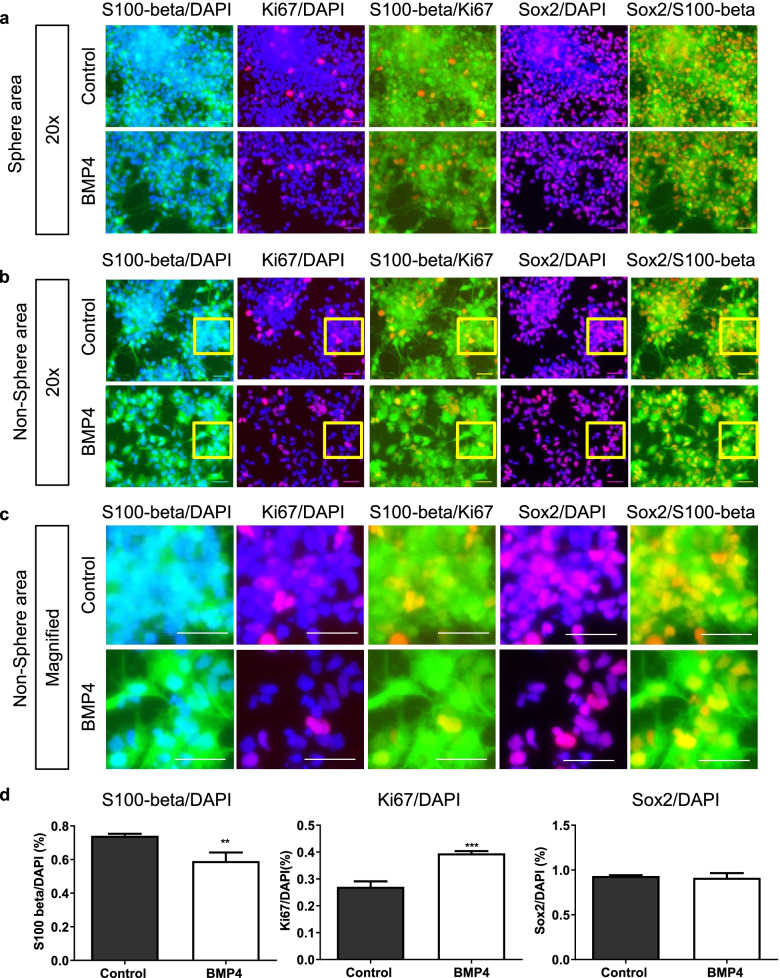
Merge images exhibits the rate of Ki67/DAPI increased, but S100-beta/DAPI decreased and Sox2/DAPI has no significant change after BMP4 treatment. **a** Merge representative images of S100-beta/DAPI, Ki67/DAPI, S100-beta/Ki67, Sox2/DAPI and Sox2/S100-beta in sphere area. **b** Merge representative images of S100-beta/DAPI, Ki67/DAPI, S100-beta/Ki67, Sox2/DAPI and Sox2/S100-beta in non-sphere area. The yellow box represents the source area of the magnified picture in (c). **c** Magnified merge images of S100-beta/DAPI, Ki67/DAPI, S100-beta/Ki67, Sox2/DAPI and Sox2/S100-beta in non-sphere area. **d** Quantitative statistics of S100-beta/DAPI, Ki67/DAPI, S100-beta/Ki67in hGSCs after BMP4 stimulation measuring by ImageJ. Data are presented as means ± SD. Student’s t-test. ∗∗*p* < 0.01, ∗∗∗*p* < 0.001

To further understand the changes in S100-beta, Ki67, and Sox2 expression ratio after BMP4 treatment, we processed and analyzed the results of immunofluorescence with their ratio to DAPI staining. The percentage of S100-beta, Ki67, and Sox2 to DAPI may indicate the cell differentiation/ cell proliferation and stemness more accurately. Firstly, we examined the area of cell ball concentration (Fig. 5a) and the area with less cell ball (Fig. 5b). Then, the local visual field was observed with magnification (Fig. 5c). Statistical analysis showed that the overall proportion of S100-beta / DAPI decreased slightly. However, the overall proportion of Ki67 increased, and the proportion of Sox2 positive cells remained unchanged compared with the absolute number after BMP4 treatment (Fig. 5d).

### Continuous stimulation enhances the inhibition of BMP4 to hGSCs

**Fig. 6:**
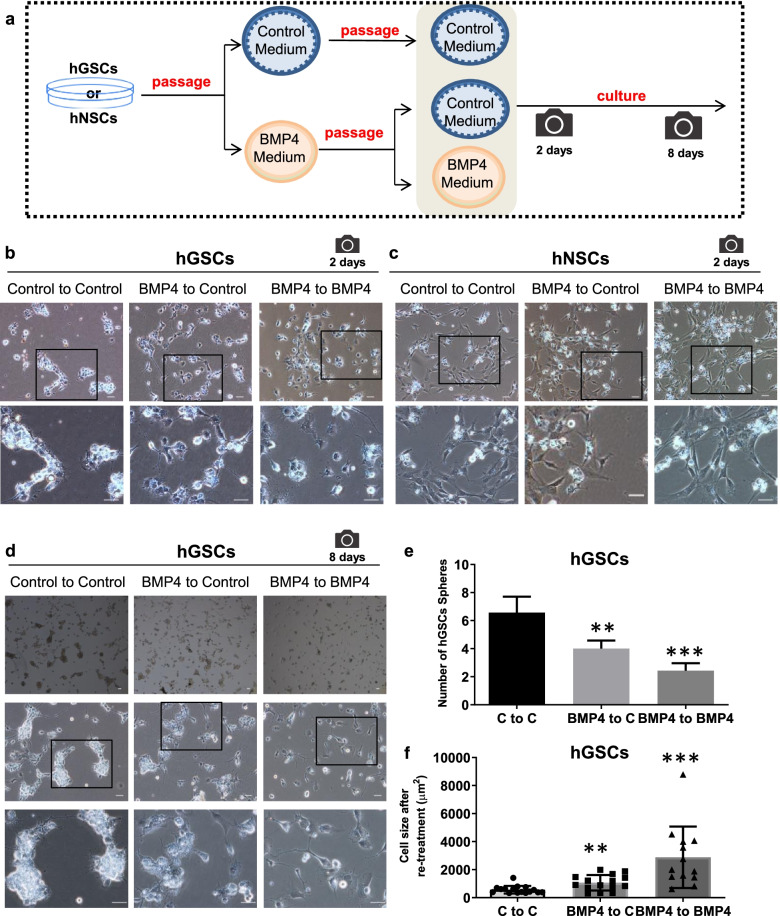
Continuous BMP4 treatment can strength the inhibition to hGSCs quantity and size, and is different from hNSCs. **a** Flowsheet of repeated exposure to BMP4 treatment and photographing time points. The effect of BMP4 continuous pressure was analyzed by the strategy of generation and repeated exposure. **b** Representative hGSCs pictures after BMP4 continuous pressure at re passage and treatment for 2 days. 0 ng/mL (Control), 20 ng/mL BMP4 (black frame indicates the second row enlarged images source). **c** Representative hNSCs pictures after BMP4 continuous pressure at re passage and treatment for 2 days. 0 ng/mL (Control), 20 ng/mL BMP4 (black frame indicates the second row enlarged images source). **d** Representative hGSCs pictures after BMP4 continuous pressure at re passage and treatment for 8 days. 0 ng/mL (Control), 20 ng/mL BMP4 (black frame indicates the third row enlarged images source). **e**-**f** Quantitative statistics of hGSCs after BMP4 repeated exposure, including number of hGSCs sphere (**e**) and Cell size (**f**) of remaining surviving clones/cells measuring by ImageJ. Scale bar, 50 μm. Data are presented as means ± SD. Student’sA t-test. ∗∗*p* < 0.01, ∗∗∗*p* < 0.001

To study the outcome of BMP4 to hGSCs and hNSCs, we designed the experiment of no stimulation and continuous stimulation after subculture (Fig. 6a). In this way, we tried to detect the effect of BMP4 on cells after continuous pressure. It was found that the number of hGSCs was significantly reduced on the day after the second passage and BMP4 stimulation (Fig. 6b). There was no significant change in hNSCs after the second passage and BMP4 stimulation (Fig. 6c). On the eighth day after the second passage and BMP4 stimulation, the number of hGSCs spherical clones cut sharply (Fig. 6d). The cell body size of adherent hGSCs amplified slightly (Fig. 6d). Statistical analysis showed that after subculture, the number of cell spheres and residual cell clones in the group with continuous additional stimulation were significantly lower than those in the control group and the single stimulation group (Fig. 6e).

## Discussions

In summary, our results revealed both the morphological and transcriptional changes after BMP4 stimulation in hGSCs and hNSCs. hNSCs exhibited larger cell size after BMP4 treatment in the first generation. In hGSCs, short-term BMP4 stimulation only inhibited cell growth and cell sphere formation but did not change the cell size. Continuous treatment of BMP4 enlarged the cell size eventually. The size of a cell may reflect the relationship between its cell cycle speed and cell metabolism level (Tzur et al. 2009). The different cell sizes lead to different reactions of animal cells to a stimulus (Ginzberg et al. 2018). Our results showed continuous stimulation of BMP4 enlarged hGSCs cell size while short-term BMP4 treatment already changed the cell size of hNSCs. Based on these observations, we suggest that hNSCs may be more sensitive to BMP4 stimulation than hGSCs. Recently study in hematopoietic stem cells (HSCs) shown that larger cell size decreased cell stemness during aging (Lengefeld et al. 2021). And the volumetric compression could induce intracellular Wnt/beta-catenin signaling in stem cells (Li et al. 2021). In our study, BMP4 changed the cell size of both hNSCs and hGSCs. The underlying molecular mechanisms are worth further validating with different time series sample collection plans.

The transcriptomic analysis suggests that BMP4 may increase glioblastoma tumor-initiating cells sensitivities (Hughes et al. 2020). However, a comparison analysis after BMP4 stimulation in both hNSCs and hGSCs is limited. Our RNA sequencing analysis identified the genes only changed in hGSCs but disorderly in hNSCs. The difference between hGSCs and hNSCs may help us to better understand the molecular mechanism of BMP4 signaling in GBM progression. The genes enriched in the cell differentiation pathway showed dual directions, which indicated the complex functional roles of BMP4 in hGSCs differentiation. In our results, a significant upregulation of astrocyte-specific marker GFAP was identified in human GSCs. The gene level of Oligodendrocyte 2 (Olig2) and the protein level of another oligodendrocyte marker, S100-beta, decreased at the same time. Similar to the role of BMP signaling in neural stem cell differentiation (Pous et al. 2020), our data confirm that BMP4 could induce glioma stem cells to differentiate into astrocytes. This functional role makes BMP4 become a popular candidate in GBM differentiation therapy (Liu et al. 2010; Piccirillo et al. 2006; Piccirillo and Vescovi 2006).

Our morphological observation indicated a substantial decreasing cell number in hGSCs. However, the pathways of cell proliferation were upregulated by BMP4 treatment. Although the total number of cells and Ki67^+^ cells reduced, the ratio of Ki67 to DAPI increased. In this case, we suggested that the underneath mechanism of how BMP4 inhibited hGSCs growth may include more than one proliferation pathway. The cell growth was significantly abrogated by BMP4, but the rest BMP4-resistant cells still exhibit high proliferation ability. Whether the inhibition of BMP4 upregulated cell proliferation genes will benefit the tumor suppressor roles of BMP4 in GBM treatment remains to be elucidated. A similar situation had been observed with Sox2 as well. After 96 hours of BMP4 stimulation, the total number of Sox2^+^ cells decreased, but the ratio of Sox2 to DAPI did not change. Sox2 is a stem cell marker of adult neurogenesis (Steiner et al. 2006). Its functional roles had been studied in both stem cells and cancer cells (Chuang et al. 2020). The high levels of Sox2 expression observed in hGSCs are indicative of their stemness. As the controversial functional role of BMP4 we introduced before, BMP4 can inhibit GSC self-renewal and tumorigenicity but cannot totally abrogate it (Sachdeva et al. 2019). Thus, it is necessary to find some partner factors working together with BMP4 to enhance the inhibition of GSCs stemness in the future.

In human GBM, BMP4 signaling downstream target ID1 was identified as a biomarker to distinguish a subpopulation of quiescent GSCs together with the opposite expression pattern of p21 in transforming growth factor-β (TGF-β) signaling (Sachdeva et al. 2019). Here, in our results, another TGF-β signaling member TGFA showed upregulation with BMP4 stimulation in hGSCs, which was not perfectly consistent with the previous finding. The different TGF-β signaling factors exhibited a dual attitude with BMP signaling agreeing with the genetic complexity of GSCs. In fact, our bioinformatic analysis in hGSC^+^/^−^hNSCs^NA^ genes showed agreement in some well-known signaling pathways mediated by BMP4, such as vascular endothelial growth factor A (VEGFA) and nuclear factor-kappa B (NF-kappa B) (Crisan et al. 2016; Wang et al. 2018). Our analysis specifically distinguished the genes diverse in hGSCs (733 genes). In clinical treatment, instead of hNSCs, we were looking for some therapeutic targets which only aim at glioma stem cells but keep the activity of normal neural stem cells. Our gene lists may help people to choose more efficient treatment methods with novel targets combining BMP4 treatment.

## Conclusions

Our study exhibited the different responses of hGSCs and hNSCs after BMP4 stimulation. BMP4 inhibited cell growth in hGSCs and enlarged the cell size in hNSCs. We used transcriptional analysis to identify gene groups only changed in hGSCs with BMP4 treatment. Cell proliferation and cell differentiation signaling pathways were enriched and consistent with the confirmation of certain protein markers in hGSCs. The top upregulated genes in our study, such as TGFA and FOXO2 could be considered as potential makers to predict the effect of BMP4 treatment on hGSCs using hNSCs-based BMP4 delivery therapy. The understanding of these different expressed genes and signaling pathways may benefit drug screening and therapy development for glioma clinical strategy.

## Methods

### Cell culture

Surgical samples and basal data were obtained in strict accordance with Ethics Committee’s permission. To establish an hGSCs line, we collected surgical samples immediately for primary culture after surgery. Briefly, the specimens were washed at least six times in 1× Hank’s balanced salt solution (HBSS, Gibco, USA). The specimens were then sheared and homogenized into small pieces using ophthalmic scissors and forceps. The tissue fragments were placed in centrifuge tubes containing 1 U/mL dispase II (Roche, USA) in 3 mL of Dulbecco’s modified Eagle’s medium (DMEM)/F12 (Gibco, USA) and incubated at 37 °C for 30 min for digestion. After digestion, the suspensions were centrifuged at 1000×g for 3 min at room temperature. The supernatants were discarded, and the tissues were suspended in 3 mL of DMEM/F12 and centrifuged again. Finally, the precipitates were resuspended in growth medium (DMEM/F12 supplemented with N-2, B-27, GlutaMAX, 20 ng/mL bFGF, 20 ng/mL EGF, 20 ng/mL heparin, penicillin, and streptomycin) on noncoated plates. Several primary glioma stem cell lines were established from different surgical samples. A cell line with multiple differentiation potential had been selected for further comparison with human neural stem cells.

An hNSCs line was established from human embryonic stem cells (hESCs) as previously described (Han et al. 2017). Briefly, human embryonic stem cells (hESCs) were pretreated with a Rho-associated protein kinase (ROCK) inhibitor (Tocris, Bristol, UK). Then, hESCs were separated from MEFs by gelatin. Nonadherent hESCs were induced by treatment with Noggin (500 ng/mL, R&D, USA) and a TGFβ inhibitor (10 mM, Tocris). The culture medium was replaced with fresh knockout serum replacement (KSR) every 2 days for 6 days. Then, the TGFβ inhibitor was removed, and the medium was replaced with 25% N2 medium and 75% KSR containing 500 ng/mL Noggin. After 2 days, the medium was replaced with 50% N2 medium and 50% KSR, and subsequently with 75% N2 medium and 25% KSR. The Noggin concentration was held at 500 ng/mL. After 10 days of induction, the freshly obtained hNSCs were cultured with pure N2 medium (DMEM/F12 supplemented with N2, GlutaMAX, FGF, EGF, heparin, penicillin, and streptomycin) for 1 day and then transferred into a pure N2/B27 medium (DMEM/F12 supplemented with N2, B27, GlutaMAX, 20 ng/mL FGF, 20 ng/mL EGF, 20 ng/mL heparin, penicillin, and streptomycin).

For hGSCs, there may exist some other types of cells in the early culture passages, cell materials from Passage 10 can ensure the purity of GSCs. For hNSCs, it is a highly pure cell line established from ES cells, but the cell differentiation ability and stemness may decrease after Passage 6. Thus, we selected Passage 4 hNSCs for the comparison experiment.

### Plates coating

For hNSC induction, 24- or 6-well plates were freshly coated with gelatin (Sigma, USA) or Matrigel (BD, USA) and hatched overnight at 4 °C. For hNSC culture and passaging, plates were precoated with poly-L-ornithine (Sigma) and laminin (Thermo Fisher, USA). The dishes for growing hNSCs were incubated with 0.5 μg/mL poly-L-ornithine in water at room temperature for at least 16 h and then washed with 1× phosphate-buffered saline (PBS). Finally, laminin (5 μg/mL) in 1× PBS was used to incubate the dishes for another 16 h. The coated dishes were stored at − 20 °C, and the supernatant was dissolved and discarded before use. All the hGSCs used in this study were seeded on noncoated plates.

### Factors

Heparin (140 mg) (Sigma, USA) was dissolved in 5.6 mL of water (25 mg/mL; 500× stock) and then filtered through a 0.22-μm filter and saved at − 80 °C. One milligram of EGF (236-EG-01 M; R&D) was dissolved in 10 mL of PBS and then filtered through a 0.22-μm filter to prepare a 5000× stock solution (100 μg/mL) and stored at − 80 °C. A 50,000× stock (1 mg/mL) of FGF (HumanZyme, USA) was prepared in 1 mL of 0.1% bovine serum albumin (BSA) and also stored at − 80 °C. Before use, FGF was first thawed and mixed and then diluted 10× with DMEM/F12. For BMP4 (HumanZyme) treatment, the protein was reconstituted in 4 mM sterile HCl containing 0.1% endotoxin-free recombinant human serum albumin. A 200× solution was stored at − 80 °C after configuration.

### Cell fixation and staining

To compare the cells in the control group and BMP4 stimulation group, we seeded the cells at the same density at the beginning of each cell line. For hNSCs, we seeded 1 million cells for 10 cm plate and 0.2 million cells for 6 wells plate. For hGSCs, we seeded 1.5 million cells for 10 cm plate and 0.3 million cells for 6 wells plate. To make sure the cells were equally separated in plates, we always suspended cells in a culture medium first and then performed cell seeding. hGSCs and hNSCs were analyzed using a staining assay similar to that described in our previous study (Han et al. 2017). Briefly, cells were cultured in an optimal condition for 3 to 7 days and then fixed with 4% paraformaldehyde for 10 to 15 min at room temperature. Later, the cells were incubated in 2.5% Triton X-100 in 1× PBS for 15 min as permeabilization. The supernatant was discarded, and the cells were blocked with 5% BSA (Solarbio, China) in 1× PBS for 1.5 h. All the procedures were performed at room temperature. The hGSCs were subsequently incubated with primary antibodies against Sox2 (goat, R&D), S100-beta (mouse, Abcam, China), or Ki67 (rabbit, Thermo Fisher) for 2 days at 4 °C. Three times washes were performed using 1× PBS containing 0.1% Tween 20 (PBST; Sigma). Distinct secondary antibodies (donkey anti-goat 633, donkey anti-mouse 488, and donkey anti-rabbit Cy3; Jackson Immuno Research, USA) were dissolved in 1 × PBS with 2.5% BSA. Secondary antibody incubation was performed for 1.5 to 2 hours at room temperature. Finally, three times washes were performed using 1 × PBST again and counterstained with 4′, 6-diamidino-2-phenylindole (DAPI; Sigma). Images were taken using an inverted fluorescence microscope (Nikon TE2000). For each immunostaining group, we performed four biological repetitions. We will randomly choose up to 5 fields for both low-power (4x) and high-power (10x and 20x) fields to make sure our statistical analyze representative.

### Sequencing and transcriptional analysis

We collected RNA samples from Passage 10 hGSCs and Passage4 hNSCs. Both hGSCs and hNSCs were treated with BMP4 for 96 hours. Two biological replicates were collected both for the control group and BMP4 treatment group. After washing once in 5 ml 1× PBS, 2 mL Trizol (Invitrogen, Carlsbad, CA, USA) was used for each 10 cm plate for genome RNA extraction. Agilent 2100 Bioanalyzer (Agilent, Palo Alto, CA, USA) was used to detect RNA integrity, and nanodrop (Thermo Fisher Scientific, Wilmington, DE, USA) was used to detect RNA quantity. Ploy(T) oligo-attached magnetic beads were used to purify poly(A)-containing mRNA. Then, cDNA synthesis was performed using an Illumina TruSeq RNA sample preparation kit (Illumina Inc., San Diego, CA, USA). The cDNA was further converted into double-stranded DNA, and AMpure XP beads were used to purify dsDNA. End-repaired and A-tailed assay was performed according to the Illumina protocol, and then PCR-amplified. HiSeq 2500 Sequencer (Illumina) was used for molecular libraries pooling and subsequently sequencing. Fragments per kilobase of transcript per million mapped reads (FPKM) values were calculated to stand for gene expression level. Pearson correlation analyses were performed by R, R- value higher than 90% indicates high consistency of the samples. The online software Morpheus (https://software.broadinstitute.org/morpheus) was used for differentially expressed gene (DEG) analysis and identified up- and down-regulated genes. Gene Ontology (GO) analyses and Kyoto Encyclopedia of Genes and Genomes (KEGG) were performed using the g:Profiler online database.

### Quantitative real time PCR (qPCR)

RNA was converted to cDNA using FastQuant RT Kit (TIANGEN, KR106). Quantitative RT-PCR was carried out in BioRad T100 PCR system using SuperRealPreMix Plus (TIANGEN, FP205) and appropriate primers. The sequences of the primers are listed below. 18 s: f-CATTCGAACGTCTGCCCTATC; r-CCTGCTGCCTTCCTTGGA; GADPH: f - TGACTCTACCCACGGCAAGTTCAA; r- ACGACATACTCAGCACCAGCATCA; BMPR2: f - ACTGCGGCTGCTTCGCAG; r - AGGCCATAGCAGGTGCTAC; Smad 1: f - GAAAGCCCTGTACTTCCTCC; r - TGAGTGGCATGTGAGGCTC; Smad 5: f - GAGAGTCCAGTCTTACCTCC; r - GTGGCATGTGTGGTTCATTG; Smad 7: f - GCTTTCAGATTCCCAACTTC; r – CTGGACACAGTAGAGCCTC. Expression levels of target genes were quantified against endogenous 18S and GAPDH levels using the comparative CT method.

### Statistical analysis

Three or more replicates were prepared and analyzed. Error bars show the standard deviation of the sample means. Statistical analysis was performed using GraphPad Prism version 8.0 (GraphPad Software, San Diego, CA, USA) and ImageJ. GraphPad Prism 7.0 for multiple comparisons. The Student’s t-test was applied to determine significant differences. Significance was assessed based on the *p*-value (*p < 0.05, **p < 0.01, and ***p < 0.001).

## Data Availability

The data and materials used in the current study are all available from the corresponding author upon reasonable request.

## Abbreviations

BMP4: Bone morphogenetic protein4
NSCs: Neural stem cells
GSCs: Glioma stem cells
hNSCs: Human NSCs
hGSCs: Human GSCs
DMEM: Dulbecco’s Modified Eagle Medium
MEF: Mouse embryonic fibroblast
TGF-β: Transforming growth factor-beta
PBS: Phosphate-buffered saline
PFA: Paraformaldehyde
FPKM: Fragments per kilobase of transcript per million mapped reads
DEG: Differentially expressed gene
KEGG: Kyoto Encyclopedia of Genes and Genomes
GO: Gene Ontology
BP: Biological process
